# A genomics-informed mechanism-based pharmacokinetic/pharmacodynamic model of cefiderocol and ceftazidime/avibactam against carbapenem-resistant *Achromobacter xylosoxidans*

**DOI:** 10.1128/aac.01263-25

**Published:** 2026-01-26

**Authors:** Bhavatharini Arun, Rajnikant Sharma, Quentin Vallé, Ngoc Minh Bui, Nicholas Furtado, María Soledad Ramirez, Gauri Rao

**Affiliations:** 1 Department of Clinical Pharmacy, USC Alfred E. Mann School of Pharmacy and Pharmaceutical Sciences, University of Southern California 5116, Los Angeles, California, USA; 2 Department of Biological Science, Center for Applied Biotechnology Studies, College of Natural Sciences and Mathematics, California State University Fullerton 14666, Fullerton, California, USA; University of Houston, Houston, Texas, USA

**Keywords:** mathematical model, β-lactams, bacterial genomics, antimicrobial resistance, *Achromobacter xylosoxidans*, immunocompromised hosts

## Abstract

*Achromobacter xylosoxidans* harbors robust intrinsic and acquired resistance mechanisms and is responsible for severe nosocomial infections in high-risk individuals. Here, we investigated the effectiveness of β-lactam antibiotic combinations against three sequentially collected *A. xylosoxidans* isolates from a pediatric patient with chronic myeloid leukemia, which were previously genotyped and sequenced to assess and characterize the evolution of resistance. The time course killing activity from *in vitro* static concentration time-kill (SCTK) assays and genomics of these longitudinally collected isolates guided the development of an *in silico* mechanistic pharmacokinetic/pharmacodynamic (PK/PD) model. As previously described, the sequentially collected *A. xylosoxidans* isolates developed resistance to meropenem and ceftazidime/avibactam during treatment, along with reduced susceptibility to cefiderocol, driven by the acquisition of β-lactamase genes, point mutations, and increased β-lactamase expression. Building on these findings, SCTK assays showed that the combination of ceftazidime/avibactam and cefiderocol achieved ≥2-log reductions in bacterial colony-forming units. The PK/PD model included two bacterial subpopulations, one resistant to ceftazidime but susceptible to cefiderocol and another resistant to both. Avibactam’s mechanistic synergy restored ceftazidime activity. However, the acquisition of resistance genes and mutations led to a 14-fold and 1.5-fold reduction in susceptibility to ceftazidime/avibactam and cefiderocol, respectively. Simulations with the developed model at clinical exposures revealed that this combination had bactericidal activity, and the infusion duration was a critical driver of efficacy. These findings underscore the therapeutic promise of combining ceftazidime/avibactam with cefiderocol for managing complex *A. xylosoxidans* bacteremia and highlight the potential of integrated mechanism-based modeling to guide treatment strategies in resistant infections.

## INTRODUCTION

*Achromobacter xylosoxidans* is a nonfermenting, aerobic, motile Gram-negative bacillus commonly found in natural environments (1). It is increasingly recognized as a significant nosocomial pathogen, particularly in immunocompromised individuals, especially those with hematological malignancies, and has been reported in numerous pediatric infection cases (2,3). While *A. xylosoxidans* can cause a wide range of clinical infections, bacteremia is the most prevalent, accounting for 96% of infections, with mortality rates reaching up to 47.5% (4, 5). In cancer patients, bacteremia poses a serious clinical challenge, often delaying chemotherapy, extending hospital stays, and complicating treatment decisions, factors that contribute to mortality rates. Moreover, immunocompromised hosts are at increased risk for developing antimicrobial resistance (AMR), which is closely linked to treatment failure and poor outcomes (6).

*A. xylosoxidans* exhibits intrinsic resistance to most currently approved antibiotics, including cephalosporins, aminoglycosides, and aztreonam. This resistance is primarily driven by multi-drug efflux systems and expression of class D β-lactamases. In addition to its innate defenses, clinical isolates frequently acquire further resistance mechanisms, making treatment increasingly complex (7). Although standardized treatment protocols for *Achromobacter* infections are not well established, β-lactams have shown promising outcomes. For instance, a study involving 10 cancer patients with *Achromobacter* bacteremia reported that all responded well to β-lactams (8). Moreover, β-lactams are frequently used in pediatric populations due to their well-established safety and tolerability profiles.

Carbapenem resistance in Gram-negative pathogens is a significant public health concern that severely limits treatment options and compromises outcomes. There are also multiple reports of carbapenem-resistant *A. xylosoxidans* strains (9). Cefiderocol, a new-generation cephalosporin, combines a catechol-type siderophore with a cephalosporin antibiotic and utilizes the siderophore–iron complex pathway to penetrate the bacterial outer membrane in addition to normal passive diffusion through membrane porins (10). Recent *in vitro* studies with cefiderocol have reported encouraging activity against *Achromobacter* spp. (11), and clinical outcomes in nine patients treated for extensively multidrug-resistant (MDR) *Achromobacter* infections have also been promising (12).

This study focuses on three sequentially collected, previously characterized *A. xylosoxidans* bloodstream isolates from a pediatric patient undergoing treatment for chronic myeloid leukemia. Following immunosuppressive treatment, the patient developed prolonged febrile neutropenia. The initial isolate was susceptible to meropenem; however, resistance emerged after 5 days of treatment. To investigate the evolution of resistance, bacterial isolates were prospectively collected and analyzed by next-generation sequencing (NGS), enabling a detailed assessment of genetic changes driving AMR that has been published previously (13).

Leveraging this unique set of longitudinal clinical isolates, this study assessed the efficacy of various β-lactam antibiotic regimens against MDR *A. xylosoxidans* isolates using a multifaceted approach. We integrated *in vitro* static concentration time-kill (SCTK) assays, NGS, and mechanism-based pharmacokinetic/pharmacodynamic (PK/PD) modeling to gain a comprehensive understanding of antibiotic killing activity and resistance evolution.

NGS provided insights into the resistance mechanisms and the evolution of AMR (13), while the SCTK assays characterized the time-dependent bacterial pharmacodynamics across various antibiotic regimens. The mechanism-based PK/PD model (MBM) was used to simulate antibiotic killing dynamics and translate *in vitro* findings to clinical relevance. By linking drug exposures achieved in pediatric patients as the driver in the MBM, we were able to design and optimize therapeutic strategies aimed at maximizing bacterial eradication while minimizing the risk of resistance development.

## RESULTS

### Assessment of pharmacodynamic activity based on *in vitro* SCTK assays linked to antibiotic resistance evolution in the patient

**Fig 1:**
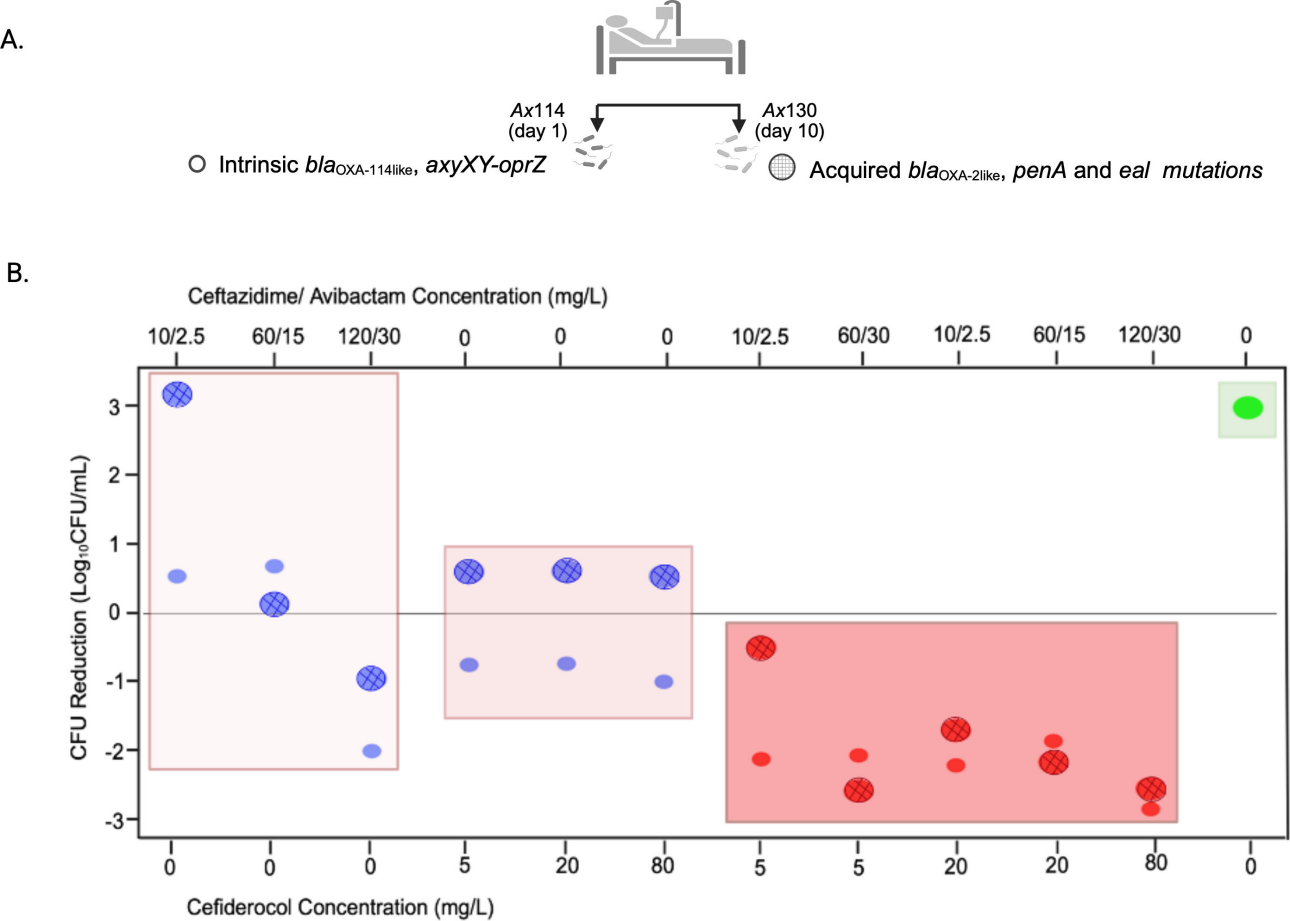
(**A**) Longitudinal *A. xylosoxidans* isolates collected from the pediatric patient. *Ax*114 (day 1) harbored intrinsic resistance genes, while *Ax*130 (day 10) acquired additional resistance genes and mutations. (**B**) CFU reduction (log_10_ CFU/mL) with ceftazidime/avibactam and cefiderocol as monotherapies (blue) and in combination (red), compared to the growth control (green). Data are shown for *Ax*114 (small circles) and *Ax*130 (larger circles with grid), highlighting differential responses across strains and treatments.

Figure 1 illustrates the concentration-response profiles at 24 h for cefiderocol and ceftazidime/avibactam monotherapies and their combination against *Ax*114, collected on day 1 of treatment, and *Ax*130, collected on day 10, using a representative range of concentrations tested in the *in vitro* SCTK experiment. Ceftazidime/avibactam monotherapy showed minimal activity, failing to achieve bacterial stasis except at the 120/30 mg/L concentration, exceeding the safety threshold of 100 mg/L for ceftazidime based on cefepime toxicity (14). This limited activity, especially against the resistant day 10 strain, aligns with the rapid emergence of antibiotic resistance in the sequentially collected *A. xylosoxidans* isolates in the pediatric patient during treatment, as previously reported (13). Initial susceptibility to meropenem was followed by increased minimum inhibitory concentrations (MICs) (up to 128 mg/L by day 10), prompting a switch to ceftazidime/avibactam, for which resistance also developed (MIC ≥ 32 mg/L). Genomic analysis revealed intrinsic resistance genes (*bla*_OXA-114-like_, *axyXY-oprZ*) in the initial strain (*Ax*114—day 1), while later isolates (*Ax*115—day 5, *Ax*130—day 10) acquired OXA-2-like β-lactamases and accumulated mutations in *penA* and *eal*. The acquired resistance mechanisms explain the reduced susceptibility to ceftazidime/avibactam (15, 16).

Cefiderocol monotherapy resulted in moderate bacterial inhibition, showing better activity against the day 1 strain with approximately a 1-log_10_ CFU/mL reduction at 24 h, consistent with the increase in MIC from 0.19 to 2 mg/L over the treatment period. Although cefiderocol was not used clinically in the patient, resistance likely developed due to the aforementioned acquired mechanisms.

In contrast, the combination of ceftazidime/avibactam and cefiderocol had the highest activity, achieving up to 2–3 log_10_ CFU/mL reduction by 24 h for both strains. Notably, in the day 10 strain (*Ax*130), the synergy between the two drugs markedly enhanced bacterial killing and effectively overcame resistance by *bla*_OXA-2_ and *penA* mutations. Additionally, no distinct concentration-dependent bactericidal activity was observed for this combination, indicating that clinically achievable exposures can effectively manage infections without requiring high doses.

Detailed results from the SCTK assays are presented in Fig. S1. Meropenem-containing combinations were relatively effective against the day 1 isolate (*Ax*114) due to its initial susceptibility. However, these regimens showed limited activity against day 5 (*Ax*115) and day 10 (*Ax*130) isolates, reflecting the emergence of meropenem resistance based on high meropenem MICs in the patient over time.

### Mechanism-based PK/PD model development and simulations

**Fig 2:**
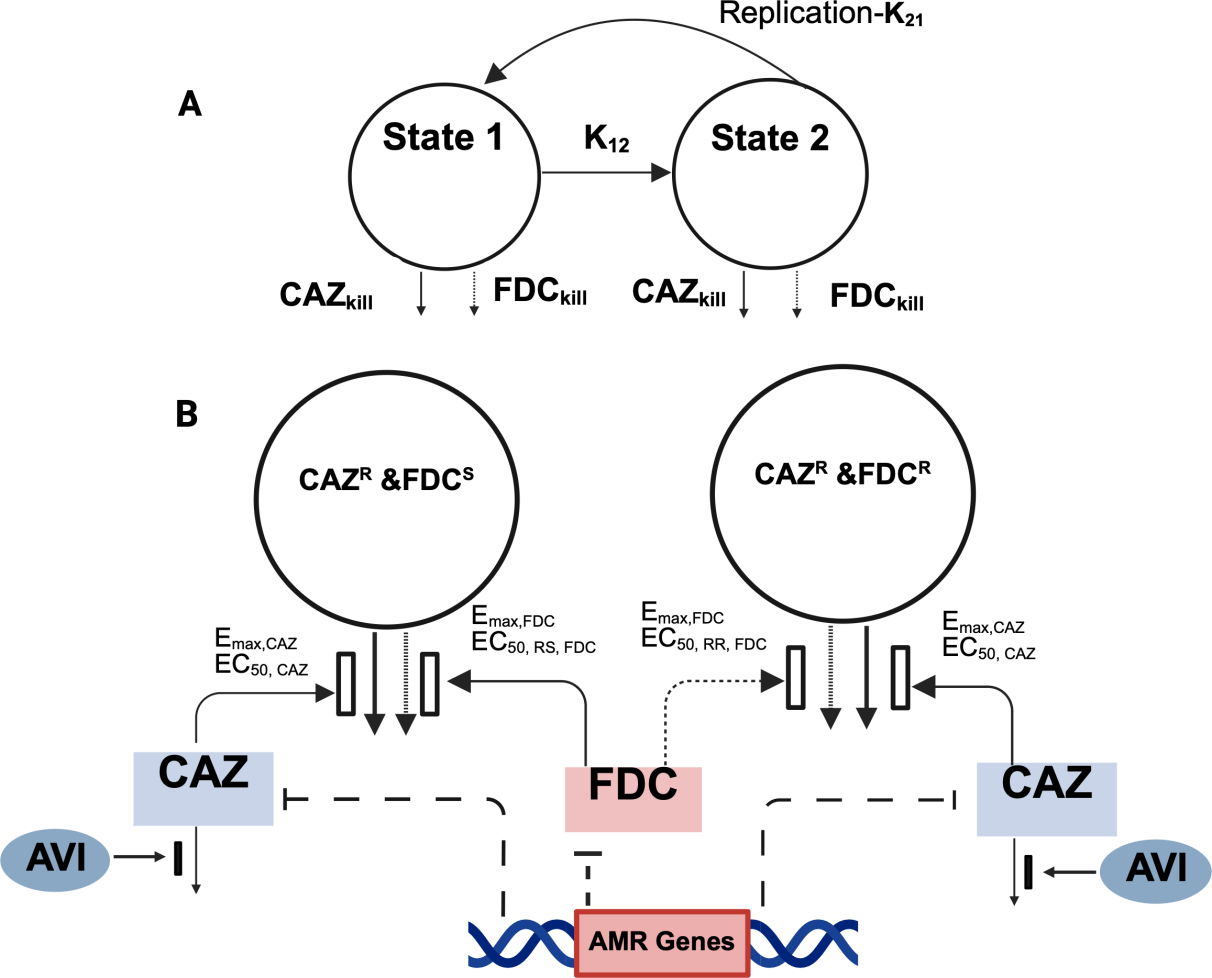
Model schematic for bacterial killing by FDC and CAZ/AVI as monotherapy and in combination. (**A**) Bacterial replication was modeled using the life-cycle growth replication model, in which each bacterial subpopulation was divided into a vegetative and replicating state. k_12_ is a first-order growth constant that controls the transition of bacteria in state 1 to state 2 for each proposed bacterial subpopulation, and k_21_ is a rapid first-order process that controls the doubling process. (**B**) A mixture model with two states in each subpopulation was used to describe the bacterial growth kinetics. The first subpopulation is FDC^S^/CAZ^R^, and the second subpopulation is FDC^R^/CAZ^R^. The parameters describing the synergistic effect of AVI on CAZ (I_max_ and IC_50, AVI_), the maximum killing rate constants (*E*_max_), and the associated antibiotic concentrations causing 50% of *E*_max_ (EC_50_) are explained in Table 1. The inhibiting effect of AMR genes on drug activity was applied to the FDC^S^ and CAZ^R^ subpopulation. FDC, cefiderocol; CAZ, ceftazidime; AVI, avibactam; AMR, antimicrobial resistance.

The mechanism-based PK/PD model, shown in Fig. 2, successfully captured the pooled SCTK data for both clinical isolates *Ax*114 and *Ax*130.

Consistent with the genotypic and phenotypic susceptibility testing, model discrimination identified two subpopulations: one resistant to both drugs and another susceptible to cefiderocol but resistant to ceftazidime, which were included in the model. The model framework assumed that cefiderocol effectively eradicated ceftazidime/avibactam-resistant subpopulations, while ceftazidime/avibactam targeted those resistant to cefiderocol, illustrating subpopulation synergy. The β-lactamase inhibition by avibactam, defined as a mechanistic synergistic term, substantially reduced the $EC_{50}$ of ceftazidime, thereby increasing the susceptibility of ceftazidime-resistant bacterial subpopulations. The model addressed the emergence of *bla*_OXA-2_ extended-spectrum β-lactamase (ESBL) genes and other mutations in the day 10 isolate (*Ax*130), characterized as a slope effect on the $EC_{50}$ of the drugs, leading to a 14-fold and 1.5-fold reduction in susceptibility to ceftazidime/avibactam and cefiderocol, respectively.

Model diagnostics confirmed the model’s precise and unbiased predictions compared to the observed viable bacterial counts for various cefiderocol and ceftazidime/avibactam concentrations tested as monotherapies and combinations *in vitro*. Detailed parameter estimates are provided in Table 1. Fig. S2 shows the model-predicted versus observed profiles for strains included in model development (*Ax*114 and *Ax*130), while Fig. S3 presents the simulated versus observed profiles for strain *Ax*115, used for model validation.

**TABLE 1:** Final parameter estimates from the mechanism-based model for the two longitudinally collected *A. xylosoxidans* isolates (*Ax*114 and *Ax*130)^*a*^

Parameter (units)	Symbol	Estimate	%RSE
Initial inoculum (log_10_ CFU)	log_10_ cfu_o_	6.57	2.41
Maximum population size (log_10_ CFU)	log_10_ cfu_MAX_	8.12	2.97
Log_10_ (mutation frequencies) for FDC^S^/CAZ^R^	log_10_ FR_SR_	0.584	25.1
Log_10_ (mutation frequencies) for FDC^R^/CAZ^R^	log_10_ FR_RR_	2.72	10.8
Bacterial doubling rate constant (h^−1^)	K_21_	50	Fix
Mean generation time for FDC^S^/CAZ^R^ (min)	K_12, SR_	111.6	10.3
Mean generation time for FDC^R^/CAZ^R^ (min)	K_12, RR_	64.14	9.93
Maximum killing rate constant of CAZ (h^−1^)	E_max, CAZ_	0.822	9.05
CAZ concentration resulting in 50% of E_max, CAZ_ in the resistant population (mg/L)	EC_50, R, CAZ_	136.9	7.65
Hill coefficient for CAZ	Hill_CAZ_	1	Fix
Maximum killing rate constant of FDC (h^−1^)	E_max, FDC_	0.530	27.3
FDC concentration resulting in 50% of E_max, FDC_ in the susceptible population (mg/L)	EC_50, S, FDC_	0.199	28.9
FDC concentration resulting in 50% of E_max, FDC_ in the resistant population (mg/L)	EC_50, R, FDC_	51.37	17.2
Hill coefficient for FDC	HILL_FDC_	1	Fix
Maximum inhibition of β-lactamases responsible for CAZ degradation by AVI	I_max_	1	Fix
AVI concentration resulting in 50% of the I_max_ (mg/L)	IC_50, AVI_	0.336	30.1
Hill coefficient for avibactam’s β-lactamase inhibition	Hill_Inh_	2.56	9.67
AMR gene effect on reducing the susceptibility to CAZ	OXA-2, _CAZ_	13.9	13.9
AMR gene effect on reducing the susceptibility to FDC	OXA-2, _FDC_	1.54	22.1
Log-additive error (log_10_ CFU/mL)	σ	0.0655	4.65

^*a*^ Th precision of each parameter is represented by the relative standard error (RSE%).

**Fig 3:**
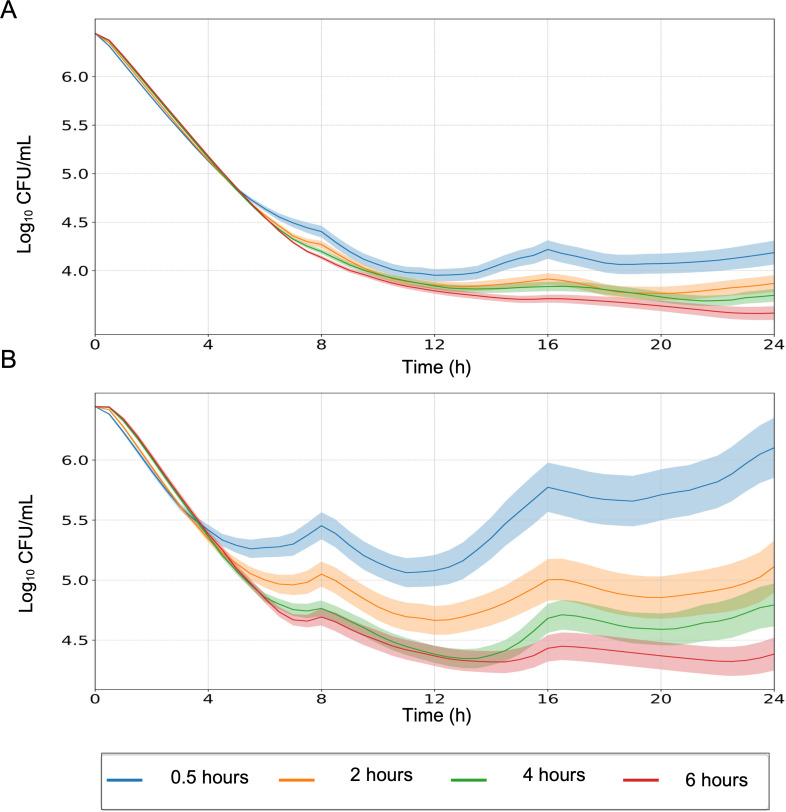
Expected *in vitro* PD effect with different infusion durations in the (**A**) *Ax*114 strain and (**B**) *Ax*130 strain. Simulations were performed using body weight-based dosing of ceftazidime/avibactam (50/12.5 mg/kg, capped at 2,000/500 mg) and cefiderocol (60 mg/kg, capped at 2,000 mg) administered every 8 h. Solid lines represent the median predicted bacterial response, and shaded areas indicate the 95% confidence interval based on the MBM simulations.

Simulations with the developed model at clinical exposures achieved in pediatric patients for two *A. xylosoxidans* clinical strains demonstrated that increasing infusion duration improved treatment efficacy, especially with the emergence of acquired resistance in the *Ax*130 isolate (Fig. 3). The simulated PK exposures for these infusion durations are presented in Fig. S4. The combined effect of cefiderocol and ceftazidime/avibactam led to a predicted 2-log_10_ CFU/mL reduction in the bacterial burden within 24 h of initiation of treatment without significant regrowth. As expected, monotherapies showed bacterial growth with no beneficial killing effect.

## DISCUSSION

*A. xylosoxidans* is a clinically challenging opportunistic pathogen, equipped with both intrinsic and acquired AMR mechanisms that enable it to evade many currently approved antibiotics. This study demonstrated the therapeutic potential of cefiderocol and ceftazidime/avibactam combination therapy against MDR clinical isolates resistant to meropenem and ceftazidime/avibactam. These isolates exhibited high-end susceptibility to cefiderocol (MIC 2 mg/L—EUCAST non-species-related breakpoint: cefiderocol MIC ≤ 2 mg/L, susceptible and >2 mg/L, resistant) (17). Genomic analysis of sequential isolates enabled modeling of resistance evolution and supported the observed effectiveness of this combination.

Cefiderocol is generally highly active against *Achromobacter* spp., with 99.1% of 228 clinical strains reported as susceptible based on EUCAST breakpoints, making it one of the most effective agents in this context. In a neutropenic murine lung infection model, cefiderocol significantly reduced bacterial burden in the lung against meropenem-resistant *Achromobacter* strains (18, 19). However, treatment of the isolate in this study was complicated by the emergence of ESBLs, such as *bla*_OXA-2_, which are known to confer resistance to ceftazidime/avibactam in *Pseudomonas aeruginosa* (20–22). The expression of these β-lactamases has also been linked to reduced cefiderocol susceptibility, and notably, there is a high cross-resistance between ceftazidime/avibactam and cefiderocol (23). For instance, cefiderocol resistance rates reached 83% among ceftazidime/avibactam-resistant KPC-producing *Enterobacterales* (24, 25). A similar trend was observed in our pediatric patient, where cefiderocol exhibited a high MIC despite no prior exposure to the drug.

The observed synergy between cefiderocol and ceftazidime/avibactam in this study is likely driven by avibactam’s inhibition of β-lactamases, which has been shown to significantly lower cefiderocol MICs, even in cefiderocol non-susceptible *A. baumannii* isolates (26). Importantly, avibactam binds to penicillin-binding protein PBP2, while cefiderocol and ceftazidime primarily bind to PBP3. This “β-lactam enhancer” action, involving simultaneous inactivation of multiple PBPs, may induce a synergistic and pleiotropic bactericidal response that extends beyond β-lactamase inhibition (27). Such mechanisms may also help prevent the emergence of cefiderocol resistance.

Our findings are supported by prior research demonstrating that the combination of cefiderocol and ceftazidime/avibactam exhibits significant *in vivo* activity against cefiderocol non-susceptible *A. baumannii* isolates, achieving complete bacterial eradication in all 12 tested isolates at humanized dosing. This combination also prevented the emergence of resistance during treatment in isolates with high-end cefiderocol susceptibility, those typically prone to resistance (26). Further investigation is needed to determine whether cefiderocol combined with avibactam alone could achieve comparable bactericidal effects.

A recent cohort study found that *Achromobacter* bloodstream infections predominantly affect immunocompromised individuals, accounting for 59.6% of cases (28). This aligns with our case, in which the patient, undergoing immunosuppressive therapy, developed prolonged febrile neutropenia. Supporting evidence from an *in vivo* study of *Acinetobacter baumannii* indicates that antibiotic exposure in neutropenic hosts accelerates AMR by fostering reservoirs of drug-resistant variants, whereas immunocompetent hosts are more effective at suppressing their emergence (6). In immunocompromised individuals, elevated bacterial burden and impaired immune responses facilitate rapid resistance development. The absence of functional phagocytic cells diminishes clonal interference, allowing resistant subpopulations to expand, even when mutations carry fitness costs.

Thus, novel approaches to guide antibiotic therapy are urgently needed in immunosuppressed populations, such as patients with febrile neutropenia undergoing intensive chemotherapy or biologic treatments. In these individuals, the frequent use of empiric broad-spectrum antibiotics can promote the selection of MDR organisms**,** increasing the risk of inappropriate initial antibiotic therapy and tripling mortality rates compared to appropriate treatment (29). Persistent infections, characterized by continuing positive blood cultures despite appropriate therapy, as observed in our patient, are associated with higher recurrence rates and poorer clinical outcomes (30).

In this study, we incorporated NGS-derived genomic evolution of *A. xylosoxidans* into the MBM, capturing resistance development through changes in drug potency (e.g., increased $EC_{50}$) during treatment. While NGS is well established in HIV care for resistance profiling and therapy optimization, its application in the management of bacterial infections is still emerging. With over 13,000 resistance genes cataloged, NGS now enables machine learning models to predict antimicrobial susceptibility with >95% accuracy for key pathogens (31). For example, a randomized trial in urologic stone surgery found that NGS-guided prophylaxis significantly reduced postoperative infections compared to standard empiric therapy. Mismatches between prescribed antibiotics and NGS results were linked to higher infection rates, underscoring the clinical value of NGS in guiding antibiotic selection (32).

Integrating NGS, MBMs, and *in vitro* systems enhances our ability to map AMR genes to clinical phenotypes and supports the transition of NGS from a diagnostic tool to a cornerstone of personalized antibiotic therapy. This integration enables real-time, evidence-based decisions regarding antibiotic selection, dosing, and infusion strategies. Multiple β-lactamase genes, including clinically significant class B metallo-β-lactamases and enzymes from classes A, C, and D, have been identified in *A. xylosoxidans* isolates. Additionally, diverse efflux pump systems, such as those from the major facilitator superfamily, multidrug and toxic compound extrusion family, small multidrug resistance family, ATP-binding cassette transporters, and resistance-nodulation-division systems contribute to its MDR phenotype (33). With the identification of additional resistance genes identified in other *A. xylosoxidans* isolates and kinetic data describing β-lactamase-catalyzed hydrolysis, the MBM framework developed in this study has the potential for expanding treatment optimization strategies across a broader spectrum of infections.

In this study, the activity of drug regimens was evaluated against only a small number of *A. xylosoxidans* isolates. Future research should include a wider range of clinical isolates with diverse resistance mechanisms to validate and refine MBM to expand its utility. Nonetheless, the availability of multiple longitudinal samples from a single patient with evolving resistance provides a valuable clinical model. The MBM can be further expanded to capture antibiotic-pathogen interactions *in vivo*, incorporating host immune responses, such as granulocyte-mediated killing, particularly relevant in immunocompromised patients. These enhancements will strengthen our findings and support clinical translation.

In conclusion, this study enhances our understanding of the genetic drivers of antibiotic resistance in *A. xylosoxidans* and highlights the therapeutic potential of combining cefiderocol with ceftazidime/avibactam to overcome resistance. By integrating multi-omics data with mechanism-based modeling, we demonstrate a potential framework for designing and optimizing targeted antibiotic combination therapies that enhance efficacy while minimizing resistance emergence.

## MATERIALS AND METHODS

### Antibiotics, medium, and bacterial isolates

Antibiotic stock solutions of meropenem (AuroMedics Pharma LLC, East Windsor, NJ), ceftazidime (Sigma-Aldrich, St. Louis, MO), and avibactam (MedChemExpress, Monmouth Junction, NJ) were prepared in 0.9% saline, while cefiderocol (MedChemExpress) was dissolved in 50% dimethyl sulfoxide. Fresh stock solutions were prepared immediately prior to each experiment and filter sterilized using a 0.22 μm Millex GP filter (Corning Inc., Corning, NY). For all *in vitro* experiments, we used freshly prepared iron-depleted cation-adjusted Mueller-Hinton broth (25.0 mg/L Ca²^+^ and 12.5 mg/L Mg²^+^, 0.75 mg/L Zn^2+^) (Difco, Detroit, MI).

Three A. *xylosoxidans* isolates, *Ax*114, *Ax*115, and *Ax*130, were collected longitudinally from a child with CML on days 1, 5, and 10 following treatment initiation(13). Meropenem, ceftazidime/avibactam, and cefiderocol MICs were determined in triplicate against each isolate using the broth microdilution method according to Clinical and Laboratory Standards Institute guidelines (34).

### *In vitro* static concentration time-kill assay

SCTK assays were conducted over 24 h to evaluate the PD activity of a range of free plasma concentrations for meropenem, ceftazidime/avibactam, and cefiderocol against the *A. xylosoxidans* isolates as previously described (35). At therapeutic doses, meropenem achieves peak serum concentrations (C_max_) of 30–71 mg/L with negligible protein binding (36). Cefiderocol has a total C_max_ ranging from 89.7 to 156 mg/L, with approximately 58% protein binding (37). For ceftazidime and avibactam, total C_max_ falls between 61.9–90.4 mg/L and 12.0–15.5 mg/L, respectively, with both drugs having protein binding of less than 10% (38). Meropenem (2, 10, 40, 120, 240 mg/L), ceftazidime/avibactam (10/2.5, 40/10, 60/15, 120/30, 240/60 mg/L), and cefiderocol (1, 2.5, 5, 10, 20, 80 mg/L) were evaluated alone and in combination against the three isolates.

While only clinically achievable unbound plasma concentrations were evaluated for cefiderocol, both clinically achievable and supratherapeutic unbound plasma concentrations were tested for ceftazidime/avibactam and meropenem monotherapies. This approach was designed based on the MICs and aimed to explore the potential benefits of high exposures to these drugs. Bacteria were quantified at 0, 1, 2, 4, 6, 8, and 24 h, with a quantification limit of 20 CFU/mL. Details of the SCTK experimental design are provided in Table S1.

### PK/PD modeling of *in vitro* SCTK assay data

An MBM was developed to describe the effects of different drug concentrations on bacterial growth and killing for the least resistant bacterial isolate collected on day 1 (*Ax*114) and the most resistant isolate collected on day 10 (*Ax*130). These phylogenetically close isolates, collected longitudinally from the same pediatric AML patient, provided genetic information that allowed for combined modeling of the two strains. The model accounted for pre-existing bacterial subpopulations with varying susceptibilities to cefiderocol and ceftazidime. Bacterial replication was modeled using a life-cycle growth model, where each subpopulation was represented in two states: vegetative and replicating. Although these states shared the same susceptibility, they differed in their growth phases.


(1) $$\text{REP}=2\;\left(1-\frac{CFU_{total}}{CFU_{max}+CFU_{total}}\right),$$

(2) $$\frac{dCFU_{1,ii}}{dt}=REP.\;K_{21}.\;CFU_{2,ii}-\;K_{12,ii}.\;CFU_{1,ii},$$

(3) $$\frac{dCFU_{2,ii}}{dt}=K_{12,ii}.\;CFU_{1,ii}-\;K_{21}.\;CFU_{2,ii}.$$

$CFU_{1}$ and $CFU_{2}$ represent the replicative and vegetative states, respectively, for each bacterial subpopulation. The transition between these states is governed by $K_{12}$, the rate at which replicative bacteria become dormant, and $K_{21}$, the rapid transition rate from dormancy to active division, fixed to 50 h^−1^. The replication factor governs the growth rate based on the total population relative to the maximum carrying capacity $CFU_{\max}$. The mean generation time $\left(\text{MT}{\text{T}}_{\text{12}}=\frac{1}{K_{12}}\right)\;$ defines the average time required for bacteria to transition from replication to dormancy. The killing effect of the drugs modeled using Hill-type equations and avibactam was modeled to enhance the killing effect of ceftazidime. The mechanistic synergy term quantifies this enhancement based on the maximum synergy ($I_{\text{max}}$) and the concentration of avibactam required for half-maximal synergy ($IC_{50}$).


(4) $$\text{Syn}=1-\frac{I_{max}.\;\left[Avi\right]}{IC_{50}+\;\left[Avi\right]}.$$

The effect of AMR genes in reducing the drug susceptibility was modeled as a slope term on the potency ($EC_{50}$) of the drugs. An additive residual error log_10_ scale was estimated for bacterial counts. The final number of pre-existing subpopulations and the effects of the drugs and AMR genes were determined based on a mechanistic understanding of drug effects and model selection based on the objective function values, diagnostic plots, and precision of parameter estimates (relative standard errors). Estimation was performed using parallelized S-ADAPT software (v1.57) with SADAPT-TRAN, utilizing the importance sampling Monte Carlo parametric expectation maximization method (pmethod = 4) (39). Once the model showed good prediction for these two isolates, it was validated using SCTK data for the isolate collected from the patient on day 5 (*Ax*115) by comparing the model simulations to the observed. This strain was not included in the initial model development but had similar AMR genes to the day 10 strain (*Ax*130). MBM codes are provided in the Supplementary Materials.

### Simulation of PD effects for clinically relevant drug exposure

Simulations were performed to assess bacterial killing dynamics for PK profiles expected in pediatric patients aged 2–12 years. A population of 100 virtual pediatric patients was generated using random sampling from CDC growth charts, providing age and sex-specific height and weight. Additional covariates, including postmenstrual age, serum creatinine, and albumin levels, were sampled from normal ranges for pediatric subjects. Creatinine clearance was calculated using the Schwartz equation. The pediatric population PK model for ceftazidime/avibactam described by Franzese et al. (38) was used to simulate ceftazidime/avibactam exposures. For cefiderocol, parameters were allometrically scaled based on the adult model by Kawaguchi et al. (37) due to the lack of a pediatric population PK model. Body weight-based dosing was applied, with ceftazidime/avibactam dosed at 50/12.5 mg/kg (capped at 2,000/500 mg) and for cefiderocol at 60 mg/kg (capped at 2,000 mg), both dosed every 8 h. Unbound drug concentrations at different infusion durations of 0.5, 2, 4, and 6 h were simulated and integrated into the developed PD model to evaluate the efficacy of these regimens. Python was used for PKPD simulations utilizing the solve_ivp function from the SciPy library for solving differential equations.

## SUPPLEMENTAL MATERIAL

Supplemental material aac.01263-25-s0001.docx Table S1; Fig. S1 to S4.

ASM does not own the copyrights to Supplemental Material that may be linked to, or accessed through, an article. The authors have granted ASM a non-exclusive, world-wide license to publish the Supplemental Material files. Please contact the corresponding author directly for reuse.
