# Supplementary material for: A genomics-informed mechanism-based pharmacokinetic/pharmacodynamic model of cefiderocol and ceftazidime/avibactam against carbapenem-resistant Achromobacter xylosoxidans
Source: Antimicrob Agents Chemother. 2026 Jan 26;70(3):e01263-25. doi: 10.1128/aac.01263-25 (PMC12959123; doi:10.1128/aac.01263-25)
Supplement: Supplemental material — Table S1; Fig. S1 to S4. [file aac.01263-25-s0001.docx]

| **Bacterial isolate** |  | **MIC (mg/L)** | | | | **Concentrations used for Static Concentration Time-kill**  **Experiments (mg/L)** | | |
| --- | --- | --- | --- | --- | --- | --- | --- | --- |
|  | **MEM** | **FDC** | **CAZ** | **AVI** | **CAZ/AVI*** | **MEM** | **FDC** | **CAZ/AVI** |
| ***Ax*114-day1** | 2 | 0.19 | >256 | >256 | 4 | Within therapeutic range | | |
| ***Ax*115 – day5** | 64 | 0.5 | >256 | >256 | 8 | 2, 10, 40 | 1, 2.5, 5, 10, 20, 80 | 10/2.5, 40/10, 60/15, |
| ***Ax*115 – day10** | 128 | 2 |  |  | 32 | Supratherapeutic levels | | |
|  |  |  | >256 | >256 |  | 120, 240 |  | 120/30, 240/60 |

**Supplementary table 1:** Static concentration time-kill experimental design.

*Avibactam was tested at a fixed concentration of 4mg/L as per Clinical and Laboratory Standards Institute (CLSI) guidelines.


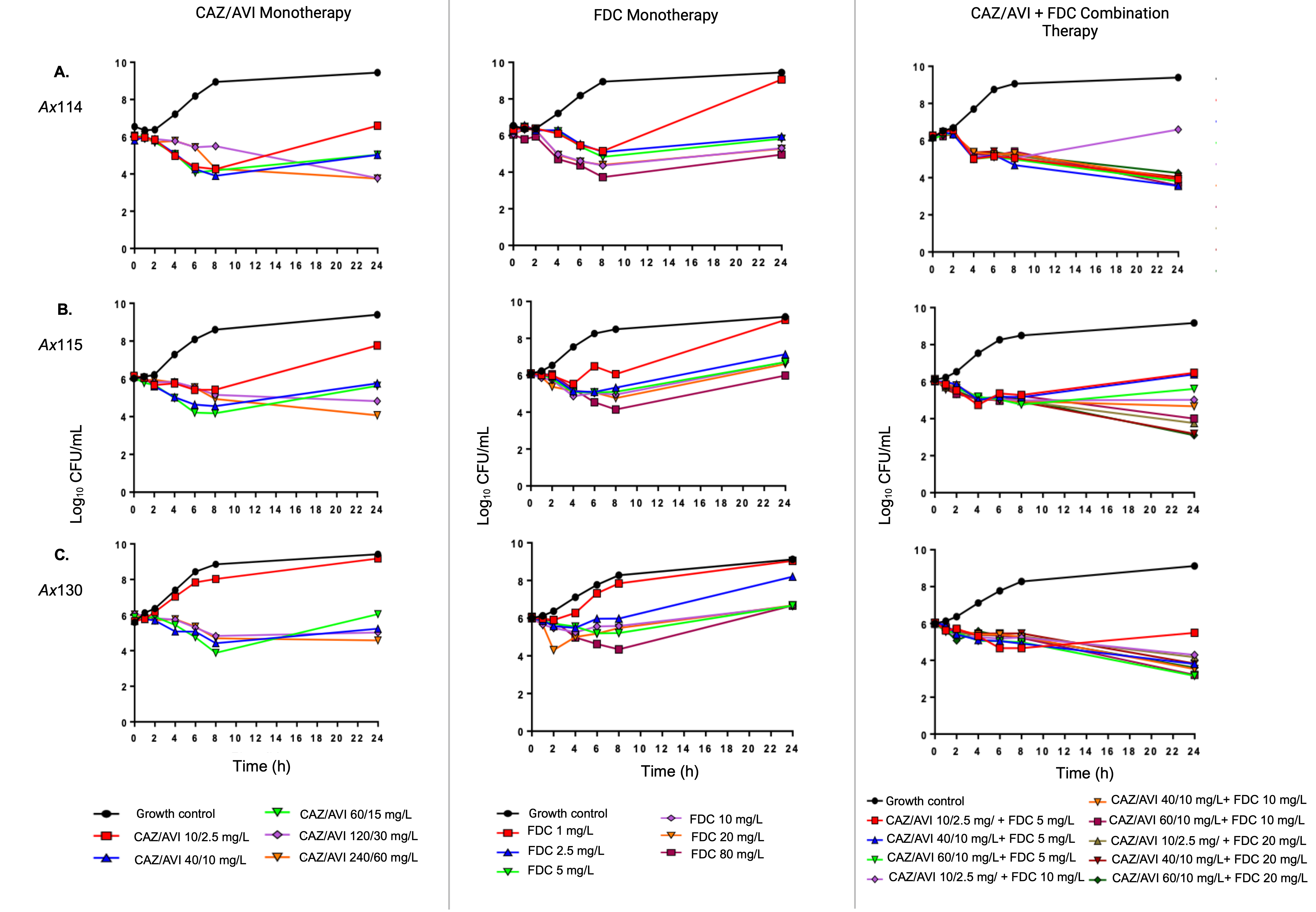


**Supplementary figure 1**: Static concentration time-kill curves illustrating bacterial burden dynamics over time. Results are shown for CAZ/AVI and fixed-dose FDC, and their combination, against three *Achromobacter* strains: (**A)***Ax*114, (**B)** *Ax*115, and (**C**) *Ax*130.


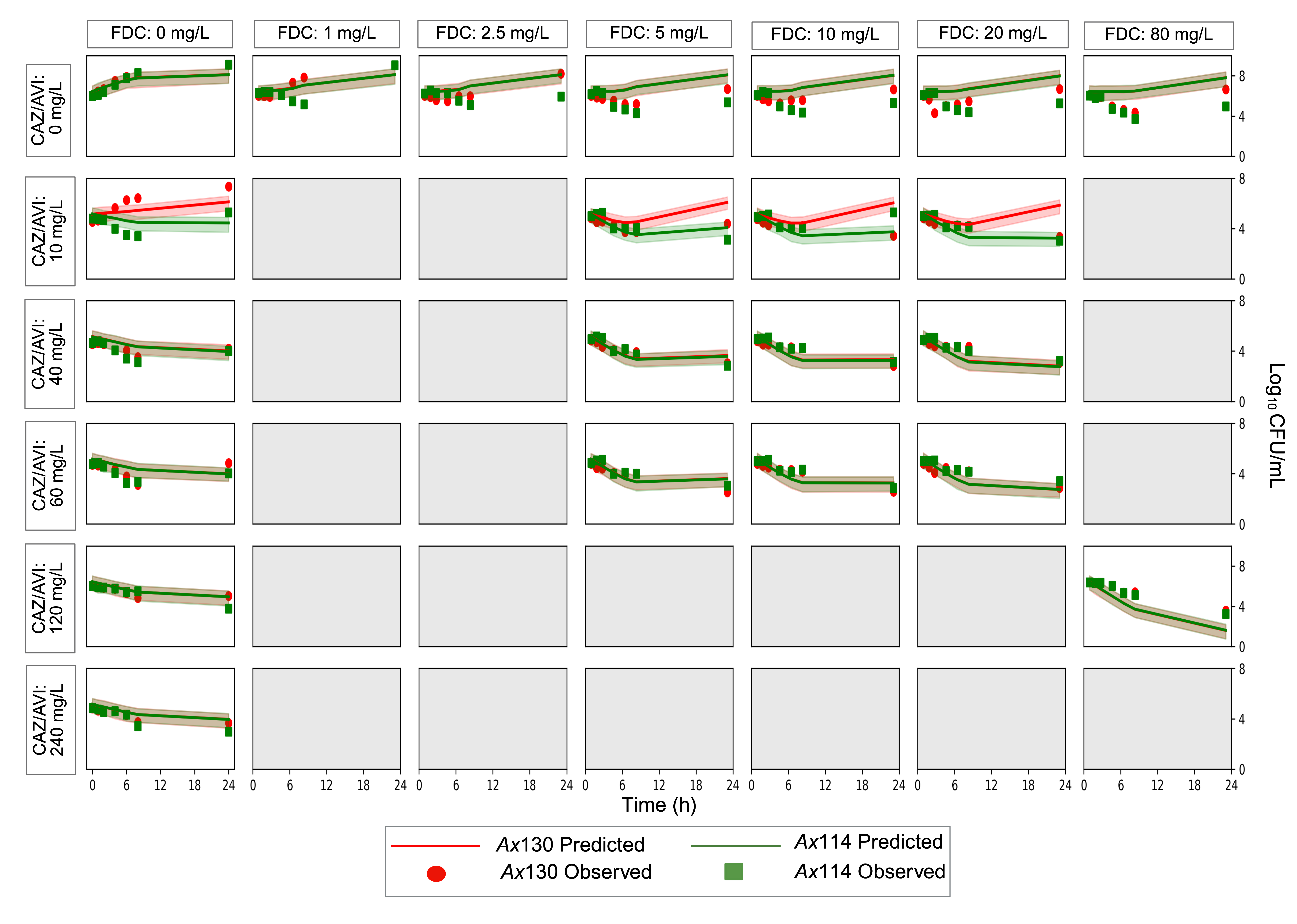


**Supplementary Figure 2A.** Simulation-based diagnostic plots showing model-predicted profiles with observed bacterial count superimposed for treatment with ceftazidime/avibactam (CAZ/AVI), cefiderocol (FDC) and their combination in *A. xylosoxidans* Ax114 and Ax130 strains included in model development. Each panel displays 1000 stochastic simulations per treatment condition. Solid lines indicate the median predicted profiles of bacterial dynamics, shaded areas denote the 95% confidence intervals, and circles represent experimentally quantified bacterial burden.


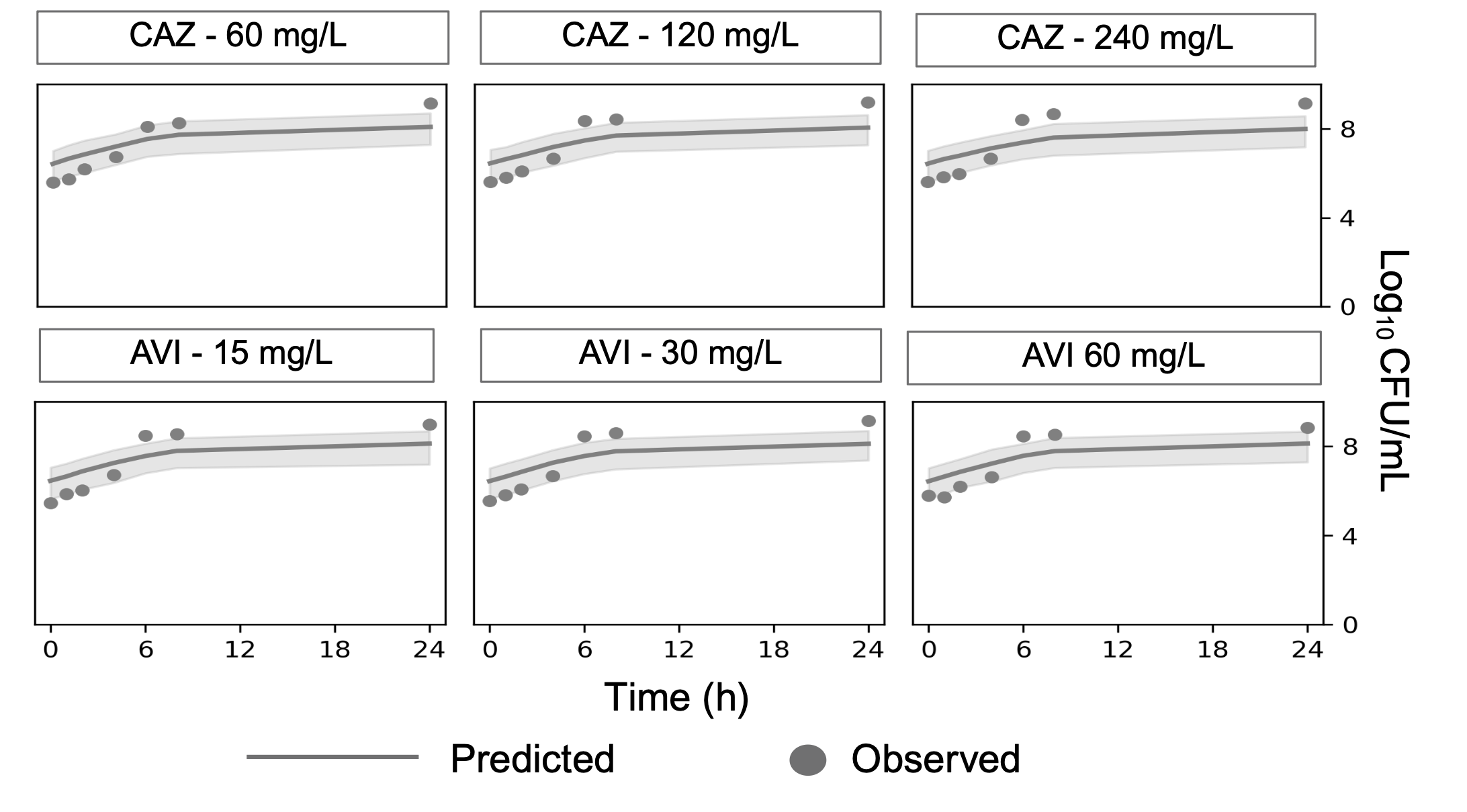


**Supplementary Figure 2B.** Simulation-based diagnostic plots showing model-predicted bacterial dynamics profiles with observed bacterial count superimposed for treatment with ceftazidime and avibactam. Solid lines indicate the median predicted profiles, shaded areas denote the 95% confidence intervals, and circles represent experimentally quantified bacterial burden.

.
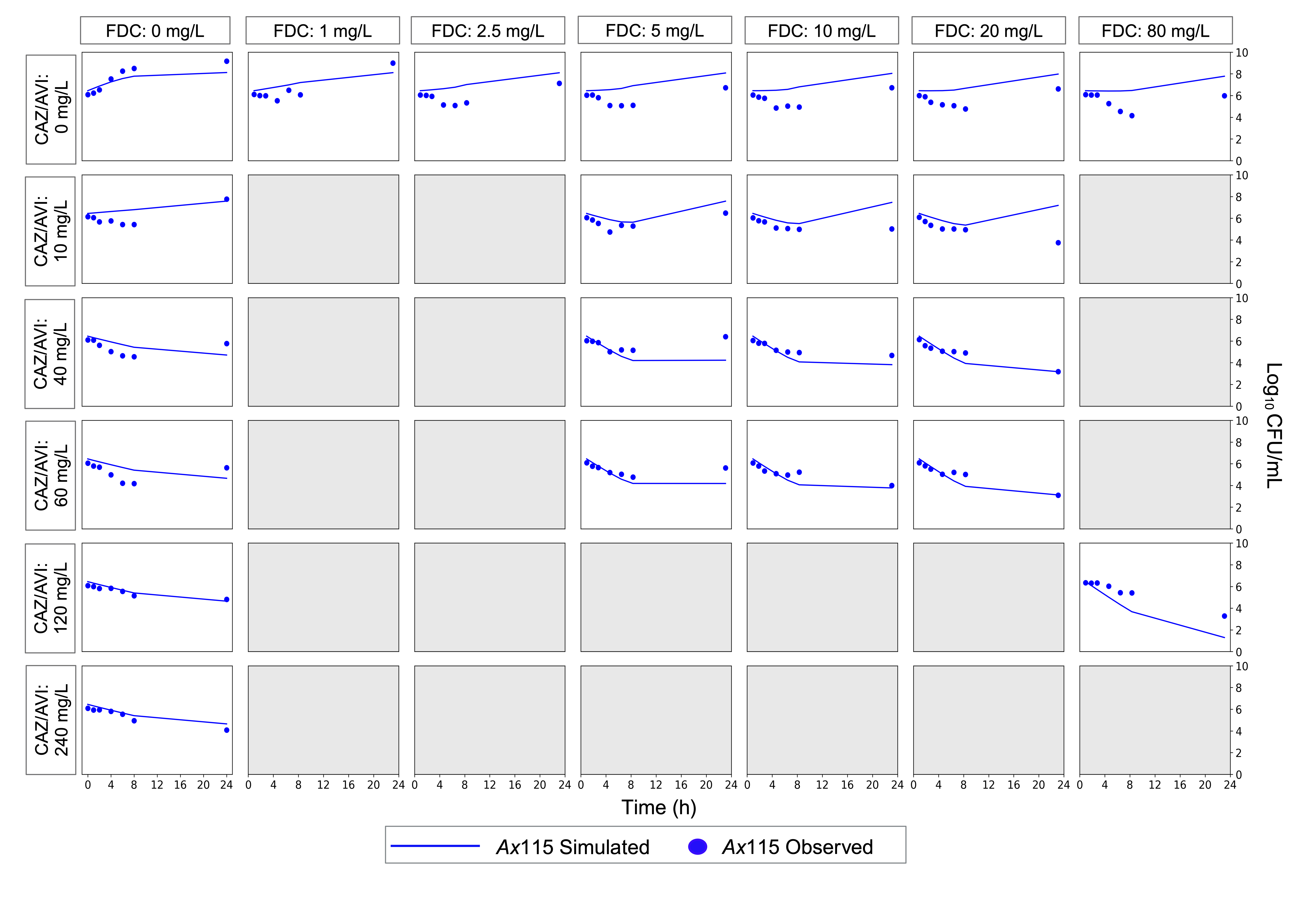


**Supplementary figure 3**: Model simulated versus observed profiles for strain *Ax*115, used for model validation.


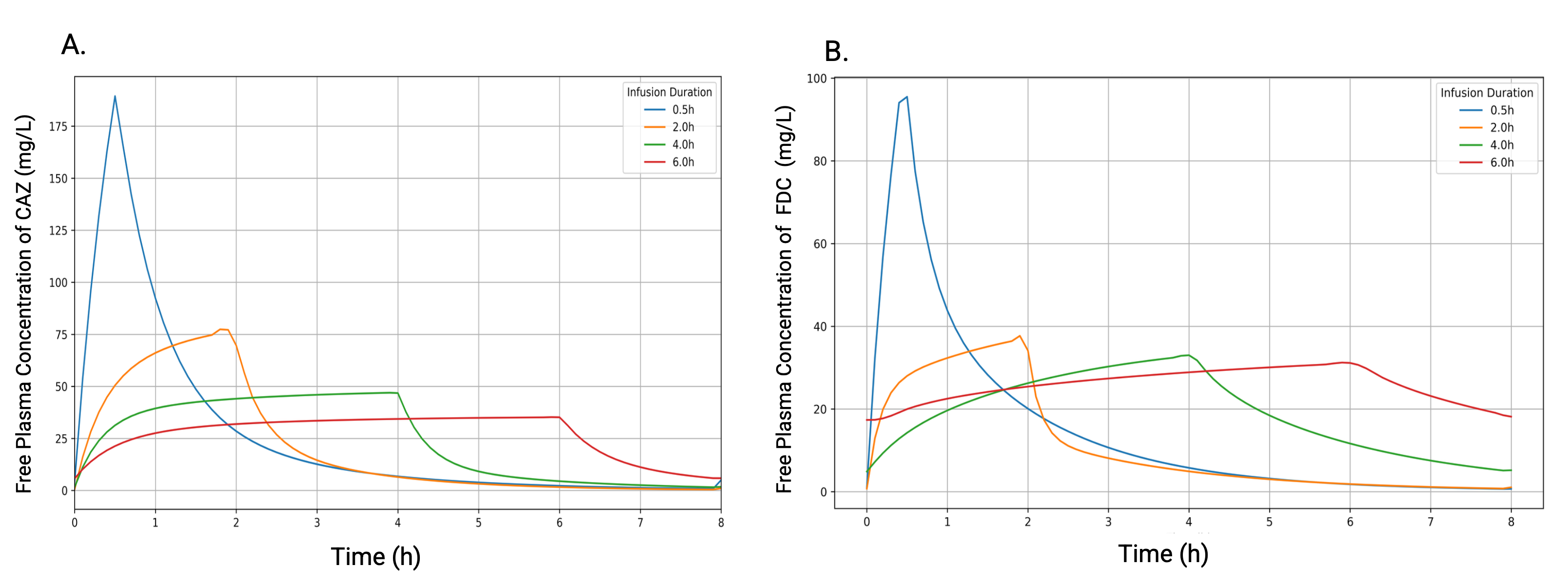


**Supplementary figure 4**: Simulated plasma concentration–time profiles for different infusion durations of ceftazidime/avibactam and cefiderocol based on the median pediatric subject from the simulation cohort.

MBM Code:

$ Ceftazidime/Avibactam (1S) AND Cefiderocol (2S)

$DIFFEQ_DIF

CFUTOT=X(1)+X(2)+X(3)+X(4)

CFUMAX = 10**(LOGCFUMAX)

REP = 2*(1-CFUTOT/(CFUMAX+CFUTOT))

IF (AVI.GT.0) THEN

SYN = 1-((IMAX*(AVI**HILL_A)) / ((IC50**HILL_A)+(AVI**HILL_A)))

ELSE

SYN = 1

ENDIF

IF (OXA2.GT.0) THEN

OXAcaz = (OXAEFF1)

OXAcfd = (OXAEFF2)

ELSE

OXAcaz = 1

OXAcfd = 1

ENDIF

IF (CAZ.GT.0) THEN

CAZR = (EMAX_C * (CAZ**HILL_C)) / (((EC50R_C * SYN * OXAcaz)**HILL_C) + (CAZ**HILL_C))

ELSE

CAZR = 0

ENDIF

IF (CFD.GT.0) THEN

CFDS = (EMAX_D * (CFD**HILL_D)) / (((EC50S_D * OXAcfd)**HILL_D) + (CFD**HILL_D))

CFDR = (EMAX_D * (CFD**HILL_D)) / (((EC50R_D)**HILL_D) + (CFD**HILL_D))

ELSE

CFDS = 0

CFDR = 0

ENDIF

K12SR = 1/(MTT12SR/60)

K12RR = 1/(MTT12RR/60)

XP(1) = REP*K21*X(2)-(K12SR+CFDS+CAZR)*X(1)

XP(2) = K12SR*X(1)-(K21+CFDS+CAZR)*X(2)

XP(3) = REP*K21*X(4)-(K12RR+CAZR+CFDR)*X(3)

XP(4) = K12RR*X(3)-(K21+CAZR+CFDR)*X(4)

$OUTPUT_ICS

CFU0=10**(LOGCFU0)

MFSR=10**(-1*LOGMFSR)

MFRR=10**(-1*LOGMFRR)

X(1)= X(1)+CFU0*(MFSR)

X(2)=0

X(3)= X(3)+CFU0*(MFRR)

X(4)=0

$OUTPUT_EQN

CFUALL=X(1)+X(2)+X(3)+X(4)

IF (CFUALL.GT.0) THEN

Y(1) = LOG10(CFUALL)

ELSE

Y(1) = 0

ENDIF

$VARMOD_EQN

V(1) = CV*CV

$POPMOD_EQN

CFD=C_CFD

CAZ=C_CAZ

AVI=C_AVI
